# Exploring the role of GIS during community health assessment problem solving: experiences of public health professionals

**DOI:** 10.1186/1476-072X-5-39

**Published:** 2006-09-18

**Authors:** Matthew Scotch, Bambang Parmanto, Cynthia S Gadd, Ravi K Sharma

**Affiliations:** 1 Department of Biomedical Informatics, University of Pittsburgh, 200 Meyran Avenue, Pittsburgh, Pennsylvania, USA; 2 Department of Health Information Management, University of Pittsburgh, 6051 Forbes Tower, Pittsburgh, Pennsylvania, USA; 3 Department of Behavioural and Community Health Sciences, University of Pittsburgh, 228 Parran Hall, Pittsburgh, Pennsylvania, USA; 4 Department of Biomedical Informatics, Vanderbilt University, 2209 Garland Avenue, Nashville, Tennessee, USA

## Abstract

**Background:**

A Community health assessment (CHA) involves the use of Geographic Information Systems (GIS) in conjunction with other software to analyze health and population data and perform numerical-spatial problem solving. There has been little research on identifying how public health professionals integrate this software during typical problem solving scenarios. A better understanding of this is needed to answer the "What" and the "How". The "What" identifies the specific software being used and the "How" explains the way they are integrated together during problem solving steps. This level of understanding will highlight the role of GIS utilization during problem solving and suggest to developers how GIS can be enhanced to better support data analysis during community health assessment.

**Results:**

An online survey was developed to identify the information technology used during CHA analysis. The tasks were broken down into steps and for our analysis these steps were categorized by action: Data Management/Access, Data Navigation, Geographic Comparison, Detection of Spatial Boundaries, Spatial Modelling, and Ranking Analysis.

27 CHA professionals completed the survey, with the majority of participants (14) being from health departments. Statistical software (e.g. SPSS) was the most popular software for all but one of the types of steps. For this step (detection of spatial boundaries), GIS was identified as the most popular technology.

**Conclusion:**

Most CHA professionals indicated they use statistical software in conjunction with GIS. The statistical software appears to drive the analysis, while GIS is used primarily for simple spatial display (and not complex spatial analysis).

This purpose of this survey was to thoroughly examine into the process of problem solving during community health assessment data analysis and to gauge how GIS is integrated with other software for this purpose. These findings suggest that GIS is used more for spatial display while other software such as statistical packages (the "What") are used to drive data management, data navigation, and data calculation (the "How"). Focusing at the level of how public health problems are solved, can shed light on how GIS technology can be enhanced to encompass a stronger role during community health assessment problem solving.

## Background

The Community health assessment (CHA) is an integral part of public health. A CHA can determine: the major health issues for a defined community, the governmental resources that need to be allocated, and the types of public health initiatives that need to be implemented. They can be conducted by: local or state health departments, universities, or private foundations. There can be many components that comprise a CHA (such as defining a geographic area of interest, establishing, and then implementing a health plan for a defined community) [1]. This paper will focus solely on the analyzing of health and population data (data analysis) phase of the assessment.

The term, "numerical-spatial problem solving" refers to performing numerical and spatial functions to analyze the health status of a geographic area (or areas) of interest [2]. This type of problem solving entails two components: a numerical component, and a spatial component. When the action to solve a problem only involves manipulation or use of numerical data (for example calculating a crude rate or summing a series of numbers), this is considered a numerical component. When the action to solve a problem only involves manipulation or use of spatial data (for example objects that have a coordinate value), this is considered a numerical component. We have previously described a numerical-spatial scenario as one that involves both spatial and numerical steps, and can be described as such:

1. Identify geographic community of interest

2. Identify health factors within the community

3. Identify bordering communities of interest

4. Identify health factors within bordering communities

5. Compare factors within community against factors of bordering community

6. Identify aggregate (state-wide, or national, etc) community

7. Identify health factors within aggregate community

8. Compare factors within community against factors of aggregate community

[2]

Use of Geographic Information Systems (GIS) can facilitate numerical-spatial problem solving. This can be seen by looking as far back as John Snow's methodology for examining the deadly Cholera outbreak in mid-nineteen century London. In order to support his hypothesis that the outbreak was caused by contaminated water, Snow combined numerical data (death counts) with spatial data (city map of London). By plotting this numerical information on the spatial information, Snow was able to identify that most of the deaths occurred in the vicinity of the Broad St. water pump. Snow obviously didn't use a GIS application to solve the problem; however, a similar scenario could be analyzed today by combining numerical information with electronic spatial data within a GIS.

GIS have been around for many years as a tool for projects such as city planning, assigning environment protection areas, and telecommunications research. It is recognized as a technology for analyzing, displaying, and manipulating spatial data. A GIS environment consists of layers (land, rivers, roads, buildings, cities, etc) on top of one another to form a detailed digital map. GIS technology is not intended to just display spatial information; it supports several powerful analysis functions such as determining best routes between two locations (network analysis), buffering (a specified distance around a particular location such as a hospital or outpatient clinic), and geo-coding (mapping coordinate points on a map such as a cohort of patients with a known environmentally induced condition). These functionalities can be very beneficial during community health assessment analysis.

There are many publications that describe the use of GIS for public health (for example [3-9]). In fact, GIS seems to be utilized a lot in environmental health analysis (for some examples see [10-16]). However, a review of the public health literature suggests an underutilization of the powerful GIS functions. Mowatt [17] indicated that while there was use (and interest) of GIS in public health, it was primarily used for simple functions such as spatial display. Chung et al [18] examined previous literature relating to the use of GIS in public health. The authors found ten relevant articles in Medline from the year 2000. They summarized that GIS is not being widely used for more advanced functions such as spatial statistical analyses but rather for simple tasks (such as geocoding and buffering) [18]. Rushton [19] highlighted GIS use in public health by type of spatial analysis. The author presents many examples where GIS has been used for complex spatial analysis as: spatial smoothing, adjusting disease rates for covariates, adjusting for social and economic deprivation, adjusting for noise, adjusting for autocorrelation, and spatial clustering [19]. While the author's literature review is a very valuable and informative, it appears as though these represent isolated incidents rather than typical GIS utilization.

There has been little information in the literature on the types of technologies used with GIS during CHA analysis. It is believed that individual software applications are used to satisfy specific steps in the numerical-spatial problem. Considering the eight steps previously outlined, a health department employee might use MS Excel to address steps two, four, five, seven, eight (numerical-related steps) and GIS for steps one, three, and six (spatial steps). There have been no instruments such as published surveys that have focused on the use of information technology (including GIS) during analysis of health and population data. A better understanding is needed to answer the "What" and the "How". The "What" identifies the specific software being used and the "How" explains the way they are integrated together during community health problem solving steps. This level of understanding will highlight the role of GIS utilization during problem solving and suggest to developers how GIS can be enhanced to take on a more prominent role during community health assessment data analysis.

This paper will address this issue by describing the results of an online survey aimed at thoroughly analyzing the process of performing community health assessment data analysis. The survey itself was designed to elicit responses on solving realistic community health assessment questions by asking participants to envision a scenario in which they have access to health and population data. Participants are then urged to describe the information technology resources within their environment that they would use to both analyze the hypothetical data and solve the hypothetical problem. The online survey was designed to capture free-text as well as short answer responses. It was believed that the different data capture formats would compliment each other. The short answer section required identification of the Information Technology (IT) used at each step in solving the question (e.g. "Microsoft Excel" for the step: *Computation of Rates*), while the free-text section allowed the participants to explain in general the different types of information technologies that they would use to answer the specific question. It was believed that creating a survey that broke down individual problem steps would highlight which types of IT are being used at different stages of numerical-spatial analysis.

## Results

Approximately 500 recruitment emails were sent out for the online survey with a total of 27 responses received (~ 5% response rate). Of the individuals deciding not to respond, some of the reasons given were: "The survey is not applicable to me", "This is survey is too technical", "I do not have the time to complete this", and "I will forward this to my colleague who will be a better person to complete it".

The authors believe that the individuals who did respond are those who highly characterize the target population of CHA professionals. The background information of the 27 respondents is shown in Table 1. All the participants who completed the survey either had performed at least one of the following activities within the last 3 years: their own community health assessment, used data from a health assessment to develop their own initiative, or commissioned a community health assessment. A valid response was any survey that was completed by someone involved with a community health assessment data in the last 3 years and thus would be able to answer "Yes" to at least one of the three background questions. The table shows the breakdown by organization.

**Table 1:** Survey participants by background

**Organization**	**Number of Survey Participants**
Health Department (local or state)	14
Other (Government agencies)	6
University	4
Healthcare Institution (Hospital, HMO, etc.)	2
Private Institution	1
Non-Government Organization	0

### Data Analysis

The data was analyzed by considering responses from both part 1 and part 2 of the survey. Part 2 was very straightforward since the responses here were one or two-word answers. Part 1, the free-text portion, was read and analyzed by the researchers to confirm or add to the responses given from part 2. In part 2, a specific software name (or names if more than one) was typically used at each step. The specific software was recorded, but for analysis purposes, the types of software were aggregated into groups. For examples, SAS, SPSS, Stata, and Epi-Info were grouped into the category of "statistical software". ArcGIS and Epi-Map (and other similar applications) were grouped into the category of "GIS software". Web resources such as online vital statistic data sets, and data analysis tools (that perform calculations) were grouped into the category "Web-based Interface". For example table 2 shows an example of a response in part 2 of the survey. Here, the response for step one is "use the state website to run queries". This response was recorded, and then grouped into the higher category "Web-based Interface". The response for the third step is "Arc GIS 9.1". This response was recorded, and then grouped into the higher category "GIS Software". The final step would add one to the grouping "Statistical Software" (for the response SAS 9.1.3) and one to the grouping "GIS Software" (for the response ArcGIS 9.1).

**Table 2:** Example response from part 2 of the survey

**Task 1**	
**Step**	**Tool(s) Used**
1. Access County-level Data	*Use state website to run queries*
2. Find deaths/100,000 in 1996	*Same as above*
3. Identify Bordering Counties	*ArcGIS 9.1*
4. Compare Bordering Counties to Allegheny County	*SAS 9.1.3 and ArcGIS 9.1*

Participant responses are in the right column. Part 2 was the driving force behind the data collection, as the free-text entry in part 1 was used to verify and confirm these responses.

All of steps in the survey were grouped into categories by the type of action they best represented. The groups were determined to be: Data Management/Access, Data Navigation, Geographic Comparison, Spatial Boundaries, Spatial Modelling, and Ranking Analysis. Each unique step was assigned an IT group that best represented it. For example, in Task one – Step one (shown in Table 2), the totals were calculated and the most frequent IT used to perform that step was "statistical software" with eleven, followed closely by "web-based interface" with eight. The most popular IT for each category is the simply the IT with the highest total. For example, "Data Management/Data Access" comprises seven of the steps in the survey. For all seven of these steps, "statistical software" is shown to be the most popular tool (with one response for web-based interface) for that category. The most popular overall for each category are shown in Table 3.

**Table 3:** Problem solving category and the most popular IT for that category

**Numerical-Spatial Problem Solving Category**	**Most Popular Type of IT**
Data Management/Access	Statistical Software
Data Navigation	Statistical Software
Geographic Comparison	Statistical Software
Spatial Boundaries	GIS Software
Spatial Modeling	Statistical Software
Ranking Analysis	Statistical Software

Participant responses are in the right column. Part 2 was the driving force behind the data collection, as the free-text entry in part 1 was used to verify and confirm these responses.

Clearly, the data indicates that statistical software is the most popular technology for most types of problem-solving purposes. GIS is the most popular for spatial boundaries (i.e. What counties border County A?).

Web-based interface tools were popular for data management/access; however it was evident from analyzing part 1 of the survey that these tools were used mostly for accessing the data while the management portion of the process would be to use a statistical software package. Thus, the responses from part 1 indicated that they used a web-based feature to view and download the data, and then used a statistical software package for the data management aspect. The focus of the example scenario was not on data access (i.e. in the scenario described on the survey, the data was already "given to them" in electronic form) but rather on the management of the data.

Since statistical software and GIS software constituted the most popular types across these categories, it was decided to examine the breakdown by specific application. This is why all individual software was recorded before being aggregated in software type. Table 4 and table 5 show the breakdown by these two different types of software. Included in with statistical software is Excel since it is commonly used in place of statistical software for numerical problem solving. The conventional map (considered as either a paper map or a map on a Web site such as Yahoo Maps) is included with the GIS software because it is a common substitute.

**Table 4:** Technology for numerical problem solving steps

**Number of Participants Using These Statistical Software Technologies**	
SPSS	11
SAS	6
Stata	3
Epi-Info	3
Excel	13

Note: A participant indicating they use both SAS and SPSS, for example, would be scored "1" for SAS" and "1" for SPSS". Included are Statistical software SPSS, SAS, Stata, and Epi-Info (other than Epi-Map) Excel is included because is a popular tool for community health problem solving and contains many basic functions found in a statistical software application. Excel was not aggregated into the category "statistical software" but is shown here because it is commonly used in place of statistical software.

**Table 5:** Technology for spatial problem solving steps.

	**Number of Participants Using These GIS Software Technologies**
ArcGIS	12
Epi-Map	1
Forestry GIS (fGis)	1
Conventional Map	8

Note: A participant indicating they use both ArcGIS and Epi-Map, for example, would be scored "1" for ArcGIS and "1" for "Epi- Map". Included are: GIS software (ArcGIS, Epi-Map, and Forestry GIS) and the conventional map, which is considered as either a paper map, or a map from a Web page such as Yahoo Maps.

Table 4 suggests that statistical software is used more than Excel (even though Excel is more popular than any tool by itself). For example, 23 out of 27 participants use statistical software (85%) where 13 out of 27 used Excel (48%) for community health assessment problem solving. Examining the individual packages, SPSS was the most popular statistical package, followed by SAS. Examining the GIS table (Table 5), GIS packages were used more often than conventional map. In fact, 14 of the 27 participants used GIS software (52%) where as eight out of 27 used a conventional map (30%). The most popular application was ArcGIS.

Table 6 shows the participants who use statistical software with other tools. In total, there were 18 participants who indicated on the survey that they use statistical software and something else while performing community health assessment data analysis. Out of those 18, 12 also use GIS software (67%). Eight of the 18 use statistical software and a Web-based interface (44%). Finally, seven out of the 18 (39%) participants use statistical software and Excel. For analysis purposes, participants could potentially use all three technologies with statistical software during a task. For example, a participant who indicated they use statistical software with GIS and also with the Web would count as "one" for GIS, and "one" for the Web.

**Table 6:** IT used with statistical software.

**Participants Who used Statistical Software with other Tools (N = 18)**	
GIS	12
Web-based Interface	8
Excel	7

*Note: A participant, who indicated they use Statistical software with GIS, and Excel, would count as "1" for GIS, and "1" for Excel*. Eighteen participants indicated in the survey that they use statistical software for community health assessment. Of the 18 individuals, 12 use GIS as well. 8 of the 18 use a Web-based interface, while 7 of the 18 used Statistical Software and Excel. The table is not summarizabile since a participant could use more than 1 type of IT.

This same type of analysis can be applied to participants who use GIS and with other tools (N = 14). This is shown in Table 7. It was already reported that 12 individuals use GIS and statistical software. Six out of the 14 who use GIS also use a Web-based interface for community health assessment problem solving (43%). Five out of 14 use Excel and GIS (36%).

**Table 7:** IT used with GIS.

	**Participants Who used GIS with other Tools (N = 14)**
Statistical Software	12
Web-based Interface	6
Excel	5

Fourteen participants indicated in the survey that they use GIS for community health assessment. It was already reported in the table above that 12 use statistical software with GIS. Of the 14, 6 use a Web-based interface with GIS and 5 out of 14 use Excel and GIS. The table is not summarizabile since a participant could use more than 1 type of IT.

### GIS and Statistical Software

The data suggests a strong relationship between GIS and statistical software. Thus if a participant is using more than one type of information technology for community health assessment analysis, it is most likely a combination of GIS and statistical software. Examining the responses specifically of the participants who use both GIS and statistical software highlights the relationship between these two technologies during numerical-spatial problem solving.

Table 8 and Table 9 are survey responses from a participant who uses statistical software and GIS for numerical-spatial problem solving. Here, the participant's responses for task 1 in both part 1 and part 2 of the survey are shown. Reviewing both parts provides an excellent description of how GIS and statistical software are used to solve numerical-spatial problems.

**Table 8:** An example survey response from part 1.

**Part 1 – Task 1**
"How does the deaths/100,000 of Allegheny County in 1996 compare to the deaths/100,000 of each of the counties that border it?"

*1. I would use ArcGIS (v9.1) to open a state map and identify the counties that border Allegheny County*.
*2. Next, using SPSS v 13.0, I would aggregate the death file and population file to get annual death totals and populations for counties by linking the geography table to the death and population tables*.
*3. Next I would compute the age-adjusted death rates for counties by year using the tables generated in the previous step (SPSS v13.0)*
*4. Finally I would generate a report using SPSS v13.0 of 1996 death rates by county after selected Allegheny and its bordering counties*.


Participant who uses both GIS and statistical software for community health analysis. Above is the participant's response (in italics) to task 1 in part 1.

**Table 9:** An example survey response from part 2.

**Part 2 – Task 1**	
"How does the deaths/100,000 of Allegheny County in 1996 compare to the deaths/100,000 of each of the counties that border it?"	

**Step**	**Tool(s) Used**
Access County level data	*SPSS v13.0*
Find deaths/100,000 in 1996	*SPSS v13.0*
Identify bordering counties	*ArcGIS v9.1*
Compare border counties to Allegheny County	*ArcGIS v9.1*

Same participant and the response (in italics) for task 1 in part 2 of the survey.

Data are loaded into SPSS for analysis. Data navigation and aggregation is done using SPSS to determine the deaths/100,000. GIS is then used for spatial display to determine the counties that border Allegheny County. Since the analysis has been done with SPSS, the participant seemingly then transfers the data into ArcView and uses spatial display to analyze the rates for Allegheny County versus its bordering areas.

This response shows how statistical software and GIS are used for numerical-spatial problem solving. Statistical software is frequently used for analysis and navigation problem solving steps and thus seems to drive the problem solving routine. GIS on the other hand, is used for simple spatial display. As CHA professionals begin to feel more comfortable with GIS technology, most likely GIS will constitute a more important component within community health assessment analysis. Health departments and universities are purchasing GIS software packages because they realize their potential. Unfortunately, end users are not utilizing their full potential; preferring to use statistical software to do the brunt of the work, and using GIS for display and reporting purposes.

## Discussion

The closest survey to the one presented in this study was done by a group in Canada in relation to information technology needs assessment and was discussed in [17]. This survey was sent to 30 community health professionals throughout Canada and was meant to gauge their interest and need for On-Line Analytical Processing (OLAP) and GIS technology during community health assessments. The survey was not designed to identify specific information technology used during numerical-spatial problem solving and how they are integrated together to solve public health questions. The survey reported that while 70% of the respondents felt that they had a good understanding of GIS, it was still being used for only simple functions [17].

Another relevant survey was developed as a result of the Turning Point program, which is a joint initiative of the Robert Wood Johnson Foundation and the W.K. Kellogg foundation [20]. One of their 5 collaboratives is the Information Technology Collaborative (ITC) whose goals is to incorporate information technology to enhance public health in the United States [21]. In 2001, the ITC commissioned a survey of local health departments to "inventory and evaluate their current use of IT and their perceived IT needs" [22] (page 125). In addition, the initiative has established an the online Public Health Toolbox as a catalog for detailing current public health systems [23]. Information only (and not access to the actual system) of the current public health tools are detailed. Examples include: BT (Bioterrorism) Sentinel, a syndromic surveillance system; MOHSIS, a system for entering, analyzing, and mapping epidemiologic information; Sudaan, a statistical software package. The systems are categorized by function (ex. Data Analysis and Mapping, Disease Surveillance, etc.) allowing the user to explore which systems are available for specific public health needs.

The Washington state department of health has established a Public Health Improvement Plan (PHIP) Information Technology Committee to focus on information needs for public health professionals and policy makers [24]. One of the tasks undertaken by the committee was to conduct a technology inventory to better understand the applications used by public health professionals across the state [24]. The survey, which is published on their website [25], does ask health officials to identity information technology used, however there is no mention of numerical-spatial problem solving tasks. The survey asks them to "list up to 10 Epidemiology, Surveillance, and Assessment custom software applications that are most useful to your LHJ [Local Health Jurisdiction] and that could be perhaps useful to another LHJ" [25]. The survey then asks the respondent to answer questions for each specific software application listed. The survey is very general in that it does not identify the IT used during the *process *of numerical-spatial problem solving. There is nothing that breaks down the steps within a numerical-spatial problem and gathers information on the IT used during these scenarios. The survey results are not publicly disclosed as per the wishes of the Washington state department of health [26].

The National Association for County and City Public Health Officials (NACCHO) and the Centres for Disease Control and Prevention (CDC) are conducting a survey to be sent to Local Public Health Agencies (LPHAs) to identify current practices [27]. The three previous studies were done in 1997, 1993, and 1990 [27]. A portion of the survey is related to community health assessment and planning. One of the questions within this section related to the use of certain community health assessment planning tools such as, Mobilizing for Action through Planning and Partnerships (MAPP), Assessment Protocol for Excellence in Public Health (APEX PH), Protocol for Assessing Community Excellence in Environmental Health (PACE EH), National Public Health Performance Standards Program (NPHPSP), and Planned Approach to Community Heath (PATCH) that that help professionals plan and organize their assessment. None of these 'tools' (these are mostly reports or planning processes) focus on information technology use during analysis of health and population data.

The NPHPSP, the National Public Health Performance Standards Program is a collaborative effort to develop performance standards for state and local public health systems [28]. Part of the initiative includes a survey sent to both local and state agencies in order to gauge their performance to the established standards. One of the topics (performance indicators) on the local public health questionnaires is "Access to Utilization of Current Technology". This indicator contains a question that asks if the health department uses GIS. Unfortunately only indicator-level results are available, thus special permission is needed for results for this specific question [29]. There is some available data that is aggregated by indicator and shows that the local public health agencies received a "30.88" compliance score (out of 100) for the indicator "Access to and Utilization of Current Technology" [30]. This score was the second lowest out of the 31 total (sub) indicator scores, suggesting significant deficiency in IT utilization among local public health agencies. The data are from 2002–2005 and represents information collected from 315 local public health systems located across the country [31].

For the response rates that could be ascertained (rates could not be determined for [24] and [28]), these surveys had higher response rates than the one described in this study (~ 5 %). This low rate was anticipated by the researchers since most of these contacts were identified through literature and Web searches. The recruitment goal prior to the study was around 30 (+/- 5). This matches the number of participants in the Canadian survey which is the most identical to our survey [17]. Braithwaite conducted a literature of online surveys to health professionals and found the range to be between 9 – 94% for 12 surveys between 1999 and 2002 [32]. It is believed that the estimated length of survey completion (20 minutes) and the requirement for free text entry related to complex problem solving scenarios might have contributed to this lower rate. Also, the fact that recruitment was via an email introduced from an unknown entity brings the potential of response-related problems such as the higher probability of the email being discarded by a spam filter or otherwise ignored by the recipient amongst a large pile of other inbox message items.

There are a few modifications to our study that, if implemented, might have achieved a higher response rate. The first is the elimination of part 1 of the survey which required free-text entry. While there was no minimum number of words required to complete the free-text portion of the survey (beyond a one-word response such as 'N/A', it is likely that some recipients declined to complete the survey after seeing the free-text boxes. Many people prefer shorter responses such as the format in part 2. Since part 2 drove the data analysis (and part 1 was used to confirm the responses), part 1 could have potentially been omitted and nearly the same results achieved. Another modification that might have resulted in a higher response rate is the use of a large public health or professional organization to 'sponsor' the survey. For example, if a local or state health department agreed to solicit the survey, then the email would be originating from an established public health organization rather than an unknown individual. This potentially could motivate recipients to complete the survey thus increasing the response rate.

### Limitations

A limitation in this study is the selection bias from the very low response rate (~ 5 %) and the convenience sampling that was used for participation of the online survey. This methodology made recruitment very easy for the researchers however also produced a low response rate that prevents the responses from being representative of public health professionals.

## Conclusion

Most CHA professionals indicated they use statistical software in conjunction with GIS. The statistical software appears to drive the analysis, while GIS is used primarily for simple spatial display (and not complex spatial analysis).

Thus the driving force behind numerical-spatial analysis in public health is statistical software and not GIS. Key components such as: data management, data navigation, and geographic comparison are done with a statistical package and then GIS is used to display a digital map of the corresponding area.

The purpose of this survey was to thoroughly examine the process of problem solving during community health assessment data analysis and to gauge how GIS is integrated with other software for this purpose. These findings suggest that GIS is used more for spatial display while other software such as statistical packages (the "What") are used to drive data management, data navigation, and data calculation ("the How"). Focusing at the problem solving level can shed light on how GIS technology can be enhanced to encompass a stronger role during community health assessment problem solving.

GIS contains statistical plug-ins than enable it to perform analytical calculations. For example, ESRI offers Geostatistical Analyst as an extension to ArcGIS [33]. The software enables for statistical analysis of geographic data including the ability to do outlier detection, prediction, and analyze global trends [33]. There are several advantages for public health researchers to use a GIS that supports statistical functionality. One is that this technology allows the researcher to perform numerical-spatial problem solving within a single application rather than utilizing a separate statistical package (and going back and forth between applications). In addition, having the ability to perform statistical calculation on geographic data extends analysis beyond the purely numerical-based functions found within a standard statistical application. It is then easier to perform public health (and geographic)-related analysis when using a combined GIS and statistical application because the data is already defined within a geographic context.

Even with powerful and useful software extensions, GIS is not being used enough. A possibility for the underutilization of GIS might be the lack of training that public health professionals have with this type of software. The considerable use of statistical packages is not surprising. There is a significant reliance on these tools for analysis in Epidemiology and public health-related research. Public health curriculums that include courses in biostatistics typically require students to utilize some sort of statistical software application for assignments. The students then learn by doing and become comfortable using these types of software. GIS, on the other hand, is not nearly as popular in public health curriculums. Only a handful of schools teach GIS courses in relation to public health. If a university does offer a GIS course, it is most likely through a geology or information and computer science department. Public health students might not feel compelled or even comfortable taking these courses outside of their domain. Public health curriculums shape the future community health assessment professionals. In order for GIS to gain in popularity, it is important for these programs to implement GIS into their programs and teach students how to use them in a hands-on manner as is done with statistical software.

Further research based on this work could be to extend the community health survey to other parts of the world such as Europe, Asia, and Africa.

## Methods

### Survey Development

The survey was electronic (accessed through the Web). The first page is the introduction page that describes the purpose of the survey, the structure of the survey, and the compensation. A link on bottom took the participant to the actual survey.

The survey itself contains four parts:

1. Background section; gathers background information on the participant such ash whether they have completed a health assessment in the last three years, and what type of organization they work at (choices included: health department, healthcare institution, university, private foundation, non-governmental organization, and other).

2. Scenario section; describes the imaginary scenario that the participants should think about when completing the survey. That is, they are given electronic data sets and are asked to analyze the health and population data by completing five community health assessment tasks.

3. Survey Part 1; describes the five tasks that they would need to solve. Below each task is a text box. The participants are asked to describe how they would complete the task given the electronic data available to them. They are urged to think about what information technology applications (software, web-based tools) they would use to solve the task.

4. Survey Part 2; describes the same five tasks from part 1. This time, the tasks are broken out into steps (determined by the researchers with the assistance of a community health expert). Next to each step is a small text box, where the participants are asked to think about what specific IT application they would use for the particular step.

5. Survey Part 3; asks the participants to note any additional IT that they did not list in the first two parts. They were given a text box to write their responses.

For assessing content validity, the survey was sent to three experts in community health assessment. They were asked to review the survey and assess whether the tasks and the nature of the survey (especially the scenario section) was appropriate for community health professionals. Overall, the experts felt the content within the survey was appropriate. Their feedback was used to create the final version of the survey.

### Data Collection

The survey was web-based and thus the participants were contacted via an email cover letter. The cover letter described the survey; the types of participants encouraged to complete the survey, the compensation amount, and the URL to access the survey. The cover letter was similar to the introduction page, but was not as detailed in describing the survey. It was decided by the researchers to send the survey to anyone at any organization that might have conducted a community health assessment, commissioned a community health assessment, or used community health data to develop their own community-based program. The preferred time frame for these activities was within the last 3 years. This time frame is identical to the one used in the NACCHO survey described earlier [27]. The survey was estimated to take approximately 20 minutes to complete (based on the completion time of one of the community health experts who analyzed the survey for content validity). For their time, participants were sent $5.

Convenience sampling was used for recruitment [34]. The researcher responsible for sending out the survey (MS) searched the Web, the literature, and utilized contact names for anyone who seemed to be associated with the process of analyzing health and population data for community health assessments. Among the types of participants who were emailed were: health department employees such as biostatisticians/epidemiologists, university researchers, industry consultants, and community health analysts within governmental organizations. Each contact was sent the email cover letter. Follow up via email was done three weeks later.

## Competing interests

The author(s) declare that they have no competing interests.

## Authors' contributions

MS and BP conceived the survey and wrote the final version of the manuscript. CSG provided expertise on survey development and critically revised the manuscript. RKS provided expertise on content validity and critically revised the manuscript.
